# Journey of water in pine cones

**DOI:** 10.1038/srep09963

**Published:** 2015-05-06

**Authors:** Kahye Song, Eunseop Yeom, Seung-Jun Seo, Kiwoong Kim, Hyejeong Kim, Jae-Hong Lim, Sang Joon Lee

**Affiliations:** 1 School of Interdisciplinary Bioscience and Bioengineering, Pohang University of Science and Technology (POSTECH), 77 Cheongam-Ro, Nam-Gu, Pohang, Gyeongbuk, 790-784, Korea; 2 Department of Mechanical Engineering, Pohang University of Science and Technology (POSTECH), 77 Cheongam-Ro, Nam-Gu, Pohang, Gyeongbuk, 790-784, Korea; 3 Industrial Technology Convergence Center, Pohang Accelerator Laboratory, Pohang University of Science and Technology (POSTECH) , 80 Jigokro-127-beongil, Nam-gu, Pohang, Gyeongbuk, 790-784, Korea

## Abstract

Pine cones fold their scales when it rains to prevent seeds from short-distance dispersal. Given that the scales of pine cones consist of nothing but dead cells, this folding motion is evidently related to structural changes. In this study, the structural characteristics of pine cones are studied on micro-/macro-scale using various imaging instruments. Raindrops fall along the outer scales to the three layers (bract scales, fibers and innermost lignified structure) of inner pine cones. However, not all the layers but only the bract scales get wet and then, most raindrops move to the inner scales. These systems reduce the amount of water used and minimize the time spent on structural changes. The result shows that the pine cones have structural advantages that could influence the efficient motion of pine cones. This study provides new insights to understand the motion of pine cones and would be used to design a novel water transport system.

Have you ever seen pine cones on a rainy day? They exhibit different shapes from regular sunny day’s pine cones. On rainy days, pine cones fold their scales to prevent seeds from spreading under humid weather whereas on a sunny dry day, these scales gape open to release the seeds^1^. This strategy promotes the survival of a species by dispersing seeds for propagation at greater distance^2^.

Through the fact that the scales of pine cones change their position on humid days, it is notable that the motion of pine cones scales is deeply related to water. In fact, plants are the representative models that exhibit some of the most elegant applications of fluid mechanics in nature^3^. For example, movement of *Mimosa pudica* is driven by a differential osmotic swelling/shrinking process with its specialized structure^4^,^5^. In addition, osmotic gradients, potassium chloride ion influxes/effluxes, sucrose and ATPase activities are activated for motion^6^,^7^,^8^,^9^,^10^. Plants generally use the complex mechanism for motion.

However, scales of pine cones consist of dead cells^1^. Thus, the mechanism of pine cones scales mobility should be passive. The passive movement is a common phenomenon in the plant kingdom. Swirling motion of wheat awns^11^, self-burial of *Erodium cicutarium* seeds^12^, transformation of pollen grain^13^ and opening of seed pods^14^ are good examples of passive movement in plant.

Then, how can they move? The passive motions in these plants are driven by the humidity (water-potential) gradient between the cells at the tissue level (sclerenchymal tissue) and the ambient air^15^. When water is absorbed/expelled in response to air humidity, the tissue expands/shrinks anisotropically in a direction perpendicular to the fibrils’ orientation^2^,^16^. Asymmetric orientation of the fibrils at the organ level converts local swelling/shrinking to a global bending movement^17^. This change occurs sensitively; with a 1% change in relative humidity at 23 °C, the coefficient of hygroscopic expansion of the fibers (0.06 ± 0.02) is significantly lower than that of sclerids (0.20 ± 0.04) in pine cones^1^. These microscopic humidity-induced strains on the cells lead to macroscopic changes^17^.

This hygromorph is very important for pine cones, because it is related to seed dispersal. Without hygromorphs, success ratio of the seed dispersal would be lower. Given that appropriate structures and systems are essential for hygromorphs, the structural advantages of pine cones are worth studying.

In this study, we investigated the interaction between water and the morphological features of pine cones. Specifically, the effects of water transport on the positional changes in scales of pine cones were systematically studied. This study provides a better understand of the structural characteristics of pine cones.

## Results

### Tracking the pine cones movements

**Figure 1:**
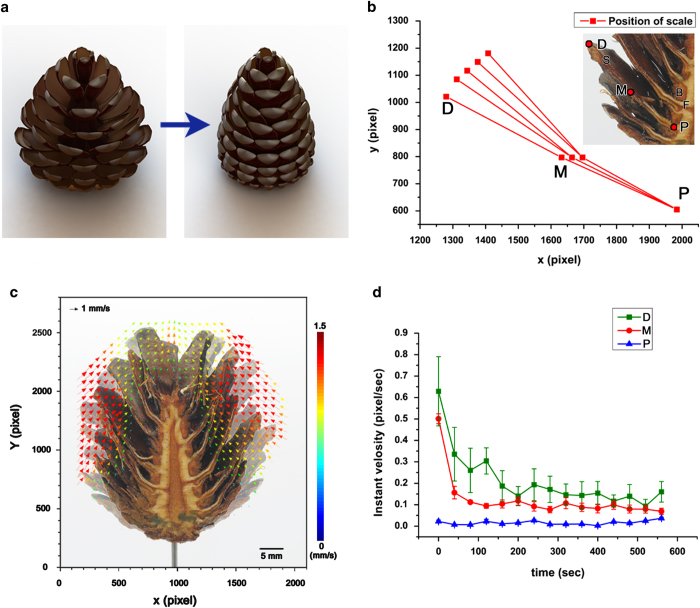
Pine cones motion in response to water (**a**) Schematic diagram of a pine cone depicts the motion of pine cone scales. When pine cones get wet, they fold their scales. The figures were created by the authors using SolidWorks software (Dassault Systèmes SolidWorks Corp., USA). (**b**) Folding trajectories of hemisected pine cones showing the distal (D), middle (M) and proximal (P) points of the scales. Symbols of ‘S’, ‘B’ and ‘F’ signify the scales, bract scales and fibers, respectively. (**c**) Displacement vectors of the scales were obtained using a PIV technique. The folding motions are mainly accentuated at the end of the scales. For clear comparison, the optical images captured at 0 and 15 min were overlapped. (**d**) Scales exhibit highest moving velocity initially, before the velocities gradually decrease. The error bars indicate the standard error (*n* = 3). Scale bar, 5 mm.

The damp pine cones close up their scales to prevent seed from releasing on humid weather^1^ (Fig. 1a). After dipping the pine cones in the water, the trajectories of folded scales were traced at intervals of 140 seconds. The positional variations of distal, middle and proximal scales were traced separately; the results are summarized as a graph (Fig. 1b). The distal point on the scale ‘D’ shows the dynamic motion that moves by 128.0 pixels in the *x*-direction and 160.0 pixels in the *y*-direction within time duration of 560 seconds. However, there was no change in the proximal point on the scale ‘P’. The displacement vectors that were obtained by applying a particle image velocimetry (PIV) algorithm to consecutive images also show a similar trend (Fig. 1c). The overlapped images exhibit the position changes of scales caused by the folding motion. The mean velocity of the distal scales is approximately 1.5 mm/s, whereas this of the proximal scales is nearly halted. Based on the result, it is concluded that motion of the pine cones caused by water transport is mainly associated with the morphological changes of the distal scales.

Temporal variation of the moving velocity at each of the scales was estimated from the displacement vectors (Fig. 1d). The distal and middle scales have maximum instant velocities of 0.6 and 0.5 pixel/sec, respectively, immediately after the pine cones were damped. Subsequently, the instant velocities of distal and middle scales decrease by 25% and 20% respectively. Meanwhile, the proximal scales maintain their original velocity at 0.0 pixel/sec.

### Route of water transport in outer pine cones

**Figure 2:**
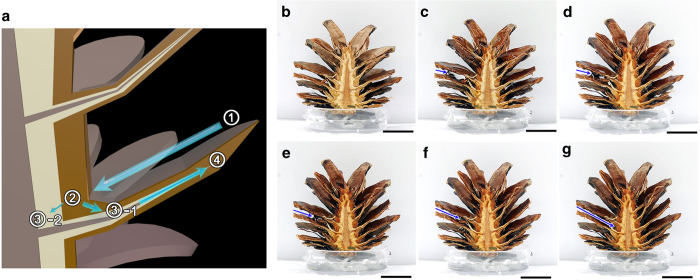
Water transport pathway in outer pine cones (**a**) Schematic diagram of a pine cone indicate the route of water transport. ① The droplet reaches the center of the pine cones ② Water is absorbed by the bract scales and spreads into the scales and fibers. ③-1 Most water is transported to the inner scales. ③-2 A small amount of water spreads to the fibers ④ The water in scales eventually causes the structural transformation. The figure was created by the authors using 3ds Max software (Autodesk Inc., USA). (**b-g**) Water droplet moves toward the center of the pine cones along the slopes of the scales and absorbed through bract scales. The blue arrows indicate the trajectories of water droplet, where the lengths of each arrow represent moving distance of the droplet. Scale bar, 4 mm.

The next question to be addressed was the mechanism by which the pine cones are damped. Consecutively captured images represent the pathway of water in pine cones (Figs. 2b–2g). Water droplets flow toward the center of the pine cones along the slopes of the scales. Blue arrows indicate the movement of a droplet. The length of the blue arrows represents moving distance from the end of the scale. This length gradually increases with the passage of time.

After the droplet reaches the center of the pine cones (Fig. 2a①), the water is absorbed by the bract scales and spreads out into the scales and fibers (Fig. 2a②). A small amount of water spreads to the fibers (Figs. 2a③–2), whereas most water is transported to the inner scales (Figs. 2a③–1), which eventually causes the structural transformation (Fig. 2a④). Since most water moves into the scales with high priority, the scales are rapidly closed up on rainy days before the whole structure gets damped. This phenomenon also signifies that small amount of water is required for the scales of pine cones to alter their morphologies. In addition, the slopes of the scales allow for the more efficient collection water toward the center of the pine cones. This is the way the pine cones close their scales rapidly.

### Route of water transport in inner pine cones

**Figure 3:**
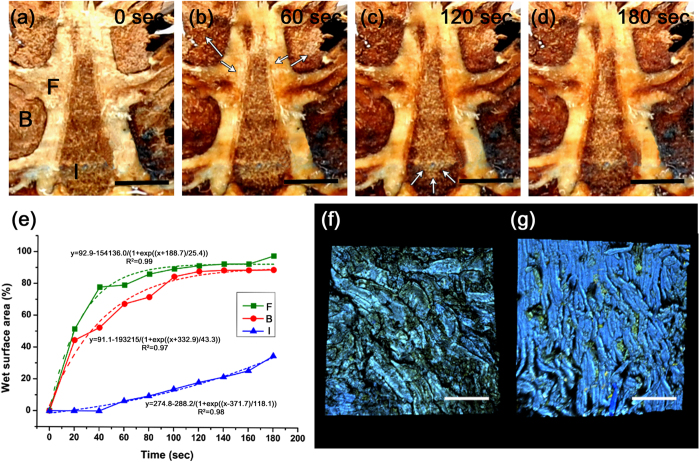
Water transport in inner pine cones (**a-d**) Water absorption in the inner pine cones was observed by dipping their bottom into water. The inner pine cones consist of three layers and they draw water up independently. White arrows represent the direction of the spreading water. ‘B’, ‘F’ and ‘I’ represents bract scales, fibers and innermost lignified structure, respectively. (**e**) Temporal variations of wet surface areas of the three layers are depicted as a graph. The time of fully damping is different each other. (**f-g**) 3D inner structures of the bract scales (**f**) and fibers (**g**) were observed using multi-photon microscopy. Their different micro-structures seemed to induce independent water uptake. Scale bars in (**a-d**) and (**f**–**g**) indicate 5 mm and 200 μm, respectively.

The mechanism by which pine cones transport most water to scales remains unclear. The secret of this system is three layer-structure of the main body of pine cones (Fig. 3). Symbols of ‘B’, ‘F’ and ‘I’ in fig. 3 represent the bract scales, fibers and innermost lignified structure, respectively. Brown bract scales and white fibers are of different materials: not only are the colors but the constructions of these layers differentiable. The bract scales have dense structures with pores of small sized (Fig. 3f). However, the threads like fibers in the middle layer are tangled up (Fig. 3g). Thus, the middle layer has relatively large pores. Detailed 3D structures are shown in Supplementary Video 1 and 2.

Above-mentioned structures induce two distinct ways of water transport. When the bract scales and the fibers are dipped in water simultaneously, the amount of water absorbed by these regions is dissimilar, namely the levels of the water rise at different pace; the bract scales and the fibers independently transport water (Figs. 3a–3d). Firstly, the water is rapidly absorbed in the boundary between bract scales and fibers (Fig. 3a). This process can be explained by preferential flow phenomenon; when water is transported through porous media with a small fracture (crack), typically through soil, most water passes through the fracture rapidly. Then the water spreads from the boundary lines into bract scales and the fibers (Fig. 3b). Figure. 3e shows that the ratios of the wetting surface area to the total surface area rapidly increase and saturate. The value of *R*^*2*^ for ‘F’, ‘B’ and ‘I’ fitting curve are 0.99, 0.97 and 0.98, respectively, which indicates that each regression curve is consistent with the experimental data for wetting process. However, wetting trends obviously differ from each other. The wet surface areas of the bract scales and the fibers sharply increase to 51.4% and 44.4%, respectively within the first 20 seconds (Fig. 3e); subsequently, the wet surface areas of the bract scales increases by 26.5% for the next 20 second-interval, whereas that of fibers increases by only 8%. However, after approximately 80% of the total area of the bract scales gets wet, the area of fibers then starts to get wet rapidly; the wet surface area of fibers increases by 14.9% at 60 second. After 100 seconds, 89.5% of the total surface area of the bract scales gets wet. Simultaneously, the fibers draw water up and approximately 88.2% are soaked by water after 120 seconds. However, innermost lignified structure does not begin to pump up water from its bottom boundary until 40 seconds elapses (Figs. 3c,3d); the innermost lignified structures slowly become wet, starting from the bottom boundary after bract scales and fibers are sufficiently soaked by water.

The average wetting rate of the bract scales and the fibers in the first 20 second-interval are 2.6 and 2.2%/sec respectively (Fig. 3e). Within 100 seconds, both the bract scales and the fibers are saturated with an average wetting rate of approximately 0.1%/sec. From this result, the water starts to be spread rapidly into the bract scales and the fibers immediately after it reaches them.

### Effects of water in pine cones scales

**Figure 4:**
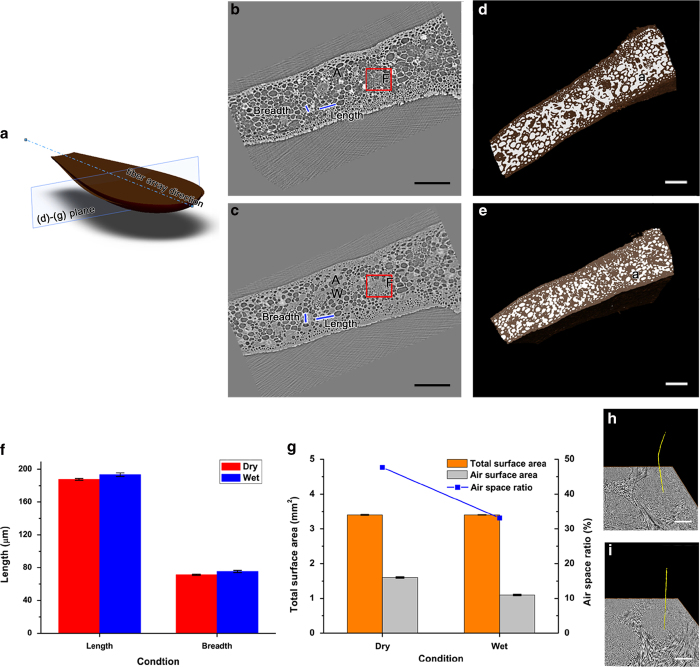
Morphological differences of inner scale under dry and wet condition (**a**) Schematic diagram of a scale indicates the direction of the fibers and measurement plane for (**b**-**e**) sections. The figure was created by the authors using SolidWorks software (Dassault Systèmes SolidWorks Corp., USA). (**b**-**c**) The inner structures of dry (**b**) and wet (**c**) scales were investigated in cross-sectional images of X-ray tomography. Symbols of ‘A’, ‘F’ and ‘W’ in X-ray images indicate air space, fibers and wet space, respectively. The air and wet spaces are clearly discriminated by image contrast. Dark gray air space turned light gray after wetting. (**d**–**e**) Sectional images were reconstructed in 3D to study their morphological changes. White color represents the air space and their volume decrease under wet condition (**e**) as compare to dry condition (**d**). (**f**) Length and breadth of the scales increase when they were completely wet. This change signifies the volume increase of the scale. (**g**) Since total surface area slightly increases while the air surface area decreases, the air space ratio consequently sharply decreases. (**h**-**i**) Morphological changes of the fiber that located at the red box in (**b**) and (**c**). The air space in the fibers perpendicular to red boxes (**b**-**c**) was labeled yellow under dry (**h**) and wet (**i**) conditions. The error bars indicate the standard deviation (*n* = 5). Scale bars in (**b-e**) and (**h**–**i**) indicate 500 and 50 μm, respectively.

Lastly, effects of water in scale were investigated. To track the events that occur in the wet scales, X-ray tomography was utilized for the comparison between the inner structures of dry scales and those of the wet scales (Figs. 4b–4e). The scales inside the outer layer have hierarchical structure with three dissimilar sizes of porous morphologies (Figs. 4b,4c). Pores of approximately 50 μm in diameter are evenly distributed near the surface of the scales. By contrast, the proximal scales have pores of approximately 150 μm in diameter. And there are fiber bundles, which have pores of approximately 10 μm in diameter. The cross sectional images were reconstructed for 3-dimensional analysis (Figs. 4d–4e,4h–4i).

Water transport through the internal structures of the scales leads to morphological changes (Fig. 4). An array of pores in fiber was labelled with yellow color in dry and wet conditions. It represents the morphological change of the fiber. When damp scales close up, bent fibers are stretched out (Figs. 4h, 4i). In addition, morphologies of inner scales are changed (Figs. 4b–4e). In dry conditions, most of the structures are filled with air, and they appear dark gray color in the X-ray images (Fig. 4b). After absorption of water, the colors of the structures turn bright gray, which indicates that most of the air spaces are occupied by water (Fig. 4c).

The morphological changes were analyzed quantitatively. The length and breadth of the wet scales increase by 3.0% (5.6 μm) and 5.5% (4.0 μm) as compared with those of the dry scales, respectively (Fig. 4f). Since air spaces filled with water, the surface area of the air spaces in wet scales are reduced by 0.5 mm^2^ (Fig. 4g). After water intake, the ratio of the air surface area to the total surface area in the scales is reduced from 47.7% to 33.1%.

## Discussion

The water absorption in pine cones represents passive actuation driven by humidity gradients; this process is investigated macroscopically and microscopically in this study. The absorption of water from the surface to the inner scales as well as the morphological changes of pine cones is investigated using multi-photon microscope and X-ray microscope with high spatial resolution. These non-destructive optical imaging tools allowed for the clear observation of the morphological structures of pine cones 3-dimensionally without any chemical treatment. In addition, the fibers are known as having an important role in the mechanism of bending by controlling the hygroscopic expansion of the cells in pine cones^1^. Thus, their morphological changes were also observed in 3-dimension (Figs. 4d–4e,4h–4i).

We found that pine cones have hierarchical structures in their inner region and scales. The three layers in the main body of the pine cones prevent water from being absorbed by the innermost layer and enhance water transport to scales with high priority. These structural characteristics are beneficial for the efficient morphological changes because they save water spending for a short period of time.

The inner scales of pine cones have two sizes of pore, excluding the pore of fibers. The large-scale pores (approximately 150 μm) are surrounded by small-scale pores (approximately 50 μm) in a sandwiches configuration (Figs. 4b–4e). The mass transport in porous media is deeply related to material properties and geometrical factors^18^. On the supposition that both porous structures are made of the same material and water pass through material surrounding pores, the difference of mass transport between the layer with small-scale pores and large-scale pores is caused by geometrical factors. Thus, effective diffusion coefficient becomes an important governing factor representing water transport ability. The effective diffusion coefficient is the important statistical parameters for porous media with the random geometry; it can be specifically applied to a bed of spherical particles^19^. The effective diffusion coefficient *D*_*eff*_ can be estimated following the relation of: 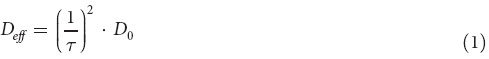 where *D_0_*is the diffusion coefficient in the free path without the presence of the obstacles^20^,^21^,^22^. The effective diffusion coefficient *D*_*eff*_ through the porous material is commonly described using the tortuosity 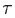 which represents the structural characteristics of the porous material. Since the most influential difference parameter between the layer with small pores and that with large pores is the porosity of the layers, tortuosity is evaluated the following model:
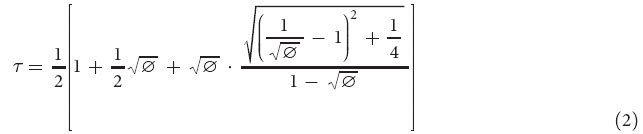
where 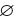 is the porosity of the material^23^. This simple geometry model explains the tortuosity of flow path in a porous material as a function of porosity. By using eqn. (1) and (2), the effective diffusion coefficient *D*_*eff*_can be estimated.

By applying the 27.4% average porosity of the layer with small-scale pores to the eqn. (1) and (2), the effective diffusion coefficient decreases by 0.69 · *D_0_*. On the other hand, when the average porosity of the layer with large-scale pores is 59.9%, the effective diffusion coefficient decreases by 0.36 · *D_0_*. The higher diffusion coefficient indicates the higher permeability of solution through the material. Therefore, a larger amount of water can be absorbed by the outer layer with small-scale pores, as compared with the inner layer with large-scale pores. Evaporation through the layered porous media is affected by thickness and sequence of layering^24^. In this aspect, scales of pine cones have an ideal structure for evaporation. If the inner layer were allowed for higher water permeability, the inner layer would decay because the inner layer is surrounded by the outer layer.

Hierarchical structures, such as that of pine cones, have received considerable attention in nature^16^,^25^,^26^. Various hierarchical combinations of structures and materials yield functional protective layers and these structures have been employed for developing new bio-inspired materials^27^. For example, nano-structures in scales of pine cones have been mimicked^17^,^28^. Unlike the previous studies, we focused on the overall water-transport system from the outer to inner scales of pine cones in a fluid-dynamics point of view, including the central region of the pine cones. In this study, we found that the hierarchical structures of pine cones are unique and beneficial for the rapid movement caused by hygroscopic expansion as well as the efficient water absorption in the scales. In addition, the hierarchical structures of scales are somehow related to water transport and evaporation. Therefore, the structural advantages of pine cones revealed in this study would provide inspiration for the development of novel, efficient bio-inspired materials or water-transport devices.

## Methods

### Estimation of movement of pine cones using PIV technique

Pine cones were cut in half using a wire saw to trace motion of a single scale without disturbance of the other scales. Optical images of the scales of pine cones during the water transport were captured using a digital camera (Nikon D700, Japan) at time intervals of 4 seconds. The captured images were analyzed using a PIV technique based on a cross-correlation algorithm for measuring the displacement of pine cones. The size of the interrogation window was fixed to 64 × 64 pixels with a 50% overlap. A recursive correlation algorithm was applied to enhance the measurement accuracy. The representative displacement between the interrogation windows was estimated by detecting the peak correlation position. The displacement was investigated at the distal, middle and proximal points were obtained (Fig. 1b). For a comparison of their instant velocities, the horizontal and vertical displacements were divided by the time interval between two consecutive images. The average moving velocities of three samples over 40 seconds were used to discern their motion trend with minimal deviation.

### Comparison of inner structure of pine cones using confocal laser scanning microscopy

Each layer of the inner structure of the hemisected pine cones was observed without any chemical treatment using confocal laser scanning microscopy (Leica Microsystems Ltd. TCS SP5 II MP with SMD, Germany) with a HC PL FLUOTAR 10 × DRY objective lens (Leica Microsystems Ltd. HC PL FLUOTAR 10 × 0.3 DRY, Germany). The Each layer was magnified using a 20 × zoomed lens. Field of view (FOV) was 775 μm × 775 μm × 408 μm; the structures were consecutively captured with 3 μm depth interval. The laser power was 3.4 kW (780 nm), and total exposure time was 185 seconds.

The acquired images were analyzed and processed using the LAS AF 2.7 software (Leica Microsystems Ltd. Germany). To improve the image quality, outlier noise was removed by filter of Image J software (National Institutes of Health, USA). The images that consecutively captured with 3 μm depth interval were reconstructed a 3D morphological structure and they were handled to make movies (Supplementary Videos 1 and 2) with Image J and Photoscape (MOOII tech, Korea).

### Water transport in inner pine cones

Hemisected pine cones were vertically placed on a petri dish with shallow water to dip bract scales and fibers simultaneously. Water uptake process was captured using a digital camera (Nikon D700, Japan) and then quantitatively analyzed using Image J software (National Institutes of Health, USA). The wet and dry surface areas were distinguished based on the difference in the image intensity. The quantitative image intensities were evaluated using image J software after enhancing images contrasts with same ratio. When the image intensity of a region of interest (ROI) is changed more than 50% compared to the initial intensity value, the ROI was assumed as a wet area; the change of intensity is caused by water absorption. After the wet area designation, the total area of wet surface was measured. The wet surface area ratios of the bract scales and fibers were depicted as a graph.

### X-ray tomography

The 3D morphological structures of the scales were observed using X-ray micro computed tomography (CT) at the 6C beamline of the Pohang Accelerator Laboratory (PAL). A 23 mm filter and a 1.5 mm-thick silicon wafer was positioned in the pathway of X-ray beam pathway to minimize the photo thermal damage to the test samples. The FOV was 4 mm × 3.5 mm and the distance from the plant sample to the camera was 20 cm. The X-ray images were consecutively collected using a sCMOS camera with 2560 × 2160 pixels (Andor Zyla, UK). The spatial resolution was 1.6 μm/pixel, which was evaluated based on the pixel size of the camera with a × 4 objective lens. The sample was fixed to a sample holder when the stage was rotated from 0° to 180° at 0.5° intervals.

Erroneous spots that appeared in the captured X-ray images were removed. A tomogram of each acquired image made to sinogram using modified Bronnikov algorithm (MBA) filter of the Octopus software (inCT, Belgium) to reconstruct with better spatial resolution^29^. The air spaces and interior structures in the reconstructed images were then labeled and quantitatively analyzed using the Amira software (Visualization Science Group, USA).

## Author Contributions

K.S. and S.J.L proposed the study. K.S., S.J.S., K.K. and J.H.L. developed and performed the experiment. K.S., E.Y., H.K. and S.J.S. analyzed the data and the processed images. All authors discussed the results. K.S. wrote the paper. K.S., E.Y. and S.J.L participated in completing the manuscript.

## Additional Information

**How to cite this article**: Song, K. *et al*. Journey of water in pine cones. *Sci. Rep.* 5, 09963; doi: 10.1038/srep09963 (2015).

## Supplementary Material

Supplementary Movie 1

Supplementary Movie 2
