# Supplementary figures and images for: Journey of water in pine cones
Source: Sci Rep. 2015 May 6;5:9963. doi: 10.1038/srep09963 (PMC4421802; doi:10.1038/srep09963)

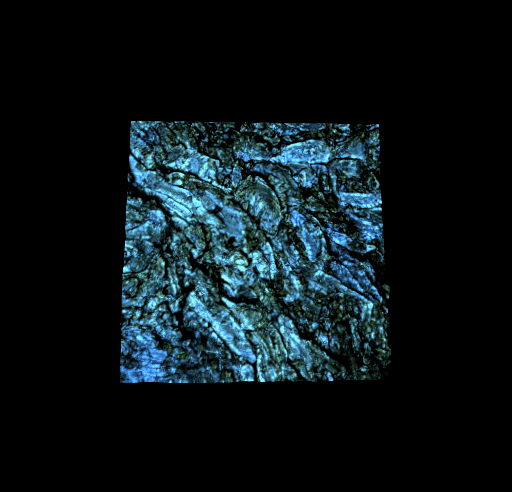

Supplement: Supplementary Movie 1 [file srep09963-s1.gif]

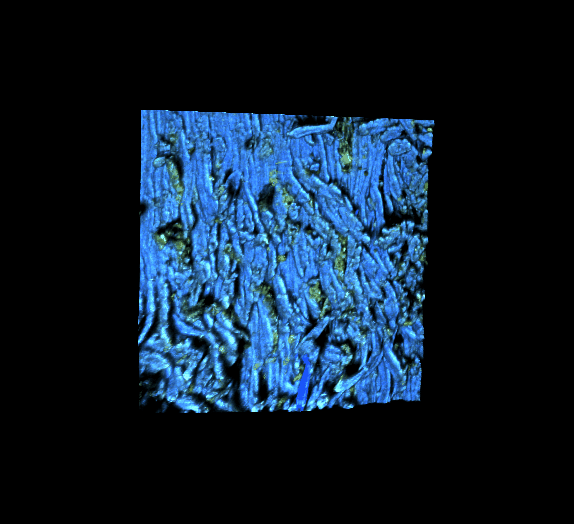

Supplement: Supplementary Movie 2 [file srep09963-s2.gif]
